# Supplementary material for: The effect of different extreme weather events on attitudes toward climate change
Source: PLoS One. 2024 May 15;19(5):e0300967. doi: 10.1371/journal.pone.0300967 (PMC11095695; doi:10.1371/journal.pone.0300967)
Supplement: S1 File — (PDF) [file pone.0300967.s001.pdf]

Online Appendix
The Effect of Different Extreme Weather Events on Attitudes Toward Climate Change

Giancarlo Visconti* Kayla Young†

February 2, 2024

Contents

A Disasters and climate change 2

B Covariates 4

C Same year 6

D Results in table format 7

*Assistant Professor, Department of Political Science, Pennsylvania State University, University Park, PA 16802, email: gvisconti@psu.edu.

†Ph.D, Department of Political Science, Purdue University, West Lafayette, IN 47907, email: young530@purdue.edu.

A Disasters and climate change

There is an emerging scientific consensus that climate change will exacerbate many types of natural disasters, making them more frequent and severe. These effects may vary between regions, however, meaning that some areas could experience more intense wildfire events while others endure extreme snow storms (Field et al., 2012). To account for this, our study includes an array of disasters that can be associated with climate change. We identity the following extreme weather events that can be associated with climate change.

Floods: While flooding can occur throughout the United States, it most often affects Northeastern and Midwestern communities. Given potential changes in precipitation because of climate change and anticipated sea-level rise, floods may become more severe and more frequent in certain regions (Field et al., 2012; Masson-Delmotte et al., 2018; NASA, 2020). Multiple studies therefore suggest a theoretical rationale for exploring the relationship between exposure to floods and attitudes about climate change (Whitmarsh, 2008; Spence et al., 2011).

Severe storms, coastal storms, and snow: Heavy precipitation, including both rain and snowfall, is believed to have already increased and will likely continue to increase in some regions due to climate change (Masson-Delmotte et al., 2018). There is evidence to suggest that the general public is attuned to these kinds of weather fluctuations (Lang, 2014). Notably, 40 percent of the US population lived in a coastal county in 2016, which are particularly vulnerable to the effects of changing precipitation compounded by sea-level rise (NOAA, 2020; IPCC, 2018).

Tornadoes: Tornadoes are a relatively frequent occurrence in the United States, appearing in the South, Midwest, and Great Plains. Data that supports the correlation between tornadoes and climate change is relatively limited (Field et al., 2012), though some scholars suggest that it may be important to consider these weather events when preparing for the risks of a changing climate (McBean, 2005).

Ice: Although data limitations similarly make understanding the potential connection between climate change and ice storms difficult, they are included in potential weather extremes analyzed by the Intergovernmental Panel on Climate Change (Field et al., 2012). Given the evidence associated with changes in precipitation patterns generally, we include extreme ice events.

Mud/landslides: (Gariano and Guzzetti, 2016, P.228) note that "different phenomena influence the stability of slopes and cause landslides, including e.g., precipitation, snow melting, temperature changes, earthquake shaking, volcanic activity, and various human actions." Therefore, while mud/landslides may not always be a direct cause of climate change, they are often associated with its anticipated alterations of the climate system. Therefore, in areas with higher precipitation, mud/landslides may be more common.

Fire: In the context of a warming planet and changes in precipitation that may lead to intense drought in some areas, fires that devastate entire towns may occur more frequently (NASA, 2020; Marlon et al., 2009). Recognizing this, some scholars have explored whether and how the public

thinks about warming, climate change, and fires (Hamilton et al., 2016).

Hurricanes: Fueled in part by warm ocean waters, hurricanes tend to affect the Southern and Eastern coasts of the United States. Major events in the past 20 years include Hurricanes Katrina, Irma, and Michael. There is general consensus that the prevalence and severity of hurricanes is connected with a changing global climate (Mann and Emanuel, 2006; Field et al., 2012; NASA, 2020). Whereas media coverage of previous decades may not have included substantial or any discussion of the potential association between such occurrences and climate change, today there seems to be more media attention to climate change after hurricanes (Cody et al., 2017).

Some disaster types that could be associated with climate change did not occur during the time period of our assessment, namely **typhoon** and **freezing** events (Field et al., 2012).[1] Similarly, **droughts** can also be connected with climate change (Field et al., 2012), but FEMA stopped reporting drought incidents since 1993. There are also federally declared disaster events that do not have clear associations with climate change and were not included here such as include: earthquakes, tsunamis, terrorist attacks, toxic substance spills, dam/levee collapses, fishing losses, chemical spills, and others (e.g., severe hardship).

Generally, **earthquakes** are not causally connected with climate change; rather, they are caused by tectonic processes not known to be correlated with global warming (Buis, 2019). **Tsunamis** are driven by earthquakes beneath or near the ocean and are not included either. While dam/levee breaks and fishing losses could both be indirectly related to climate change (e.g., Hurricane Katrina was the catalyst for the breaking of a levee in the New Orleans area that caused severe damage), it is much harder to associate these events with global warming. This is also true for disasters that are human-caused, such as chemical incidents. These events are, therefore, not included in our treatment indicator(s).

Of the nine disasters we identify, there are five that are not common enough to be able to identify an effect using the dynamic DID, these are ice storms, mud/landslides, snow storms, tornadoes, fires, and coastal storms. Consequently, we pay attention to the most common ones: severe storms, fires, hurricanes, and floods. In fact, almost nine out of ten extreme weather events associated with climate change correspond to one of these four disasters.

[1] Guam and Puerto Rico were affected by typhoons but we do not include those territories in our sample.

B Covariates

In this section, we also estimate dynamic difference-in-differences, including placebo covariates (age, gender, and education in 2010). Placebo covariates are subjects' characteristics that should not be affected by exposure to a disaster. The conclusions of the study do not change when using covariates in the DiD.

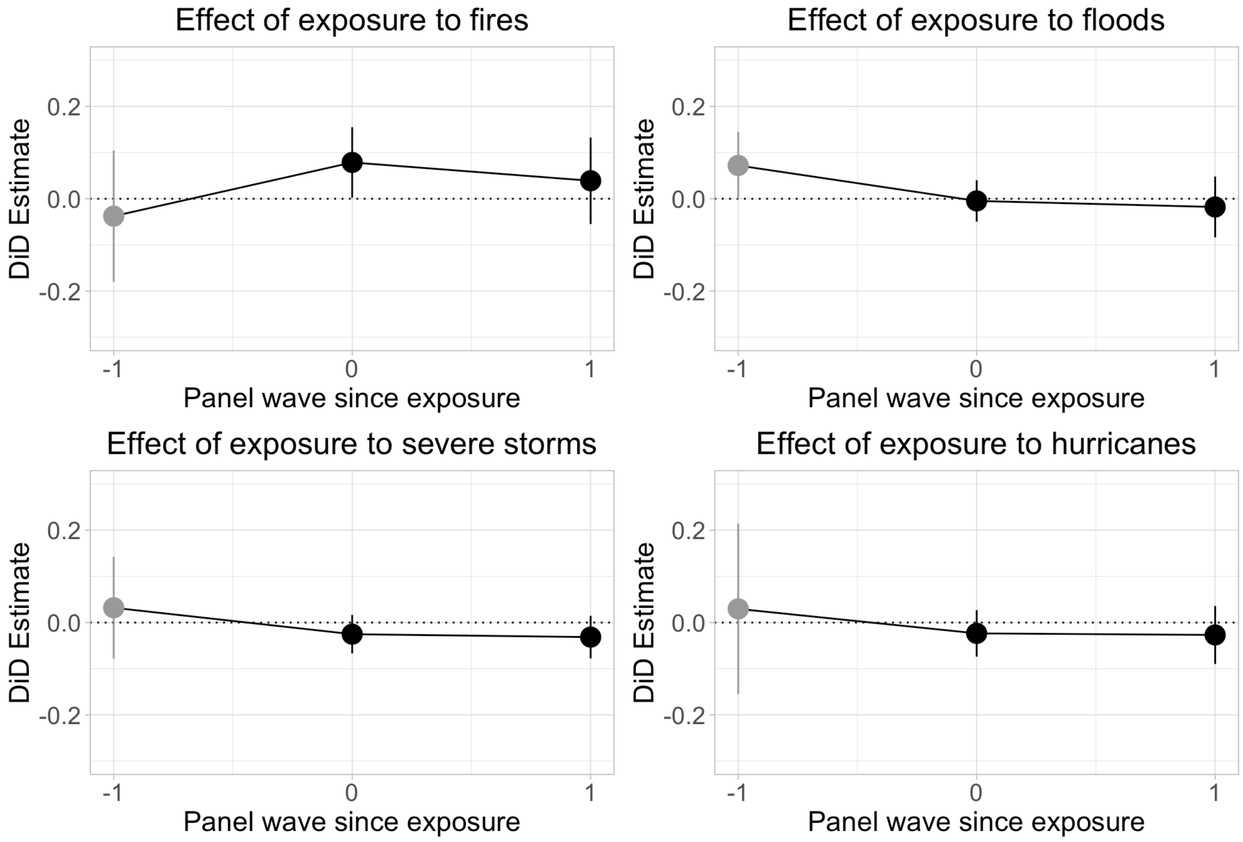

Figure A1: Effect of exposure to disasters on attitudes toward climate change (using placebo covariates). A length of exposure equal to 0 corresponds to the first or initial exposure to a disaster. A length of exposure equal to 1 corresponds to the first period after the initial exposure to a disaster. Conversely, a length of exposure equal to -1 refers to the first period before initial exposure. N = 28,128 (respondents-wave).

Also, we use partisanship as a covariate. However, this might not necessarily be a placebo covariate since exposure to disaster can affect different political outcomes, therefore, we include party ID in a different analysis. The conclusions of the study do not change when using partisanship in the DiD.

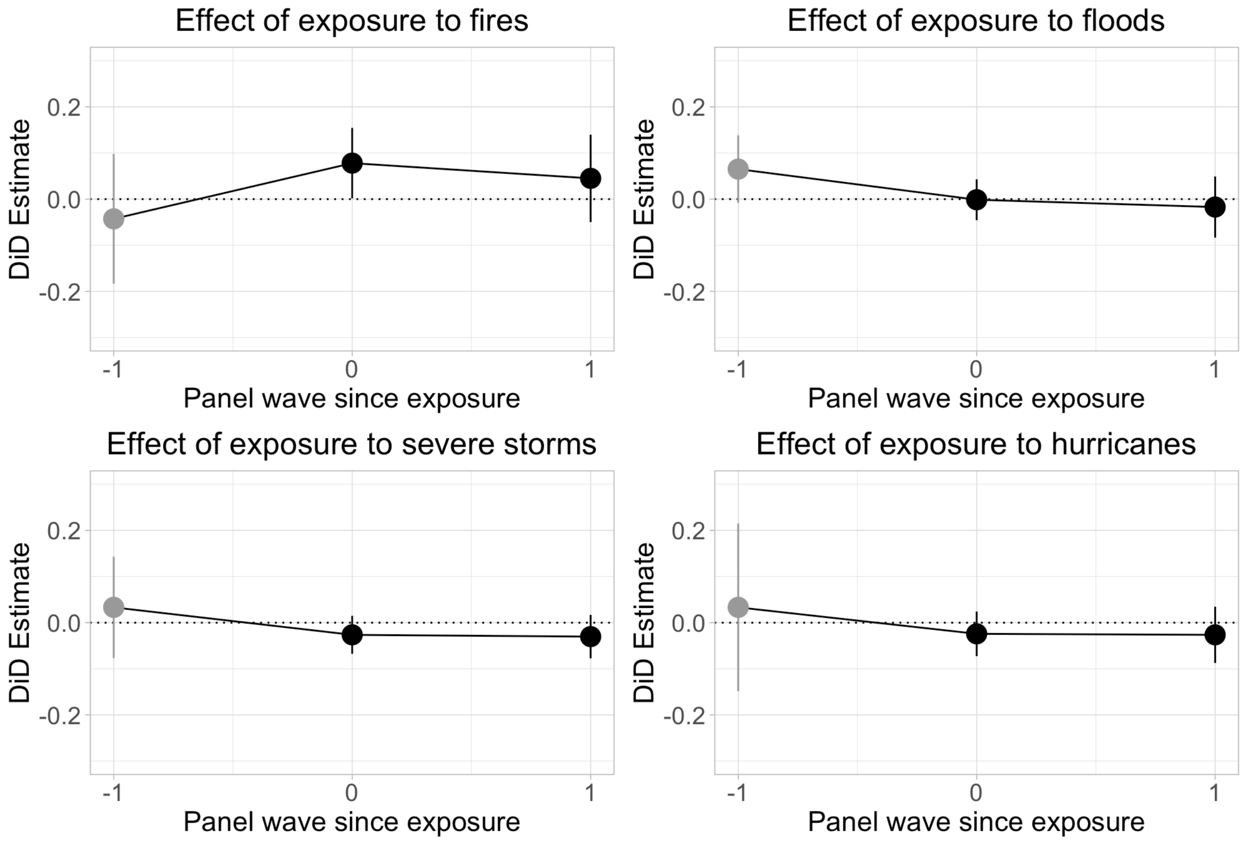

Figure A2: Effect of exposure to disasters on attitudes toward climate change (using partisanship). A length of exposure equal to 0 corresponds to the first or initial exposure to a disaster. A length of exposure equal to 1 corresponds to the first period after the initial exposure to a disaster. Conversely, a length of exposure equal to -1 refers to the first period before initial exposure. N = 28,128 (respondents-wave).

C Same year

Our exposure indicator measures a given disaster happening in the year before implementing a given panel survey wave. As a robustness check, we constructive an alternative indicator of whether a given disaster happened the same year of the panel survey wave implementation but before it was conducted in October of that year. In this analysis, we could not obtain results for hurricanes because of the lack of pre-treatment periods to test. In the case of the other three disasters, we found consistent evidence with our main results. Floods or severe storms have no impact on concerns about climate change. In the case of fire, the plot shows a null finding, but we observe a significant increase when estimating the overall effect (combining all the post-treatment periods), with an increase in 0.062 points (p-value lower than 0.1).

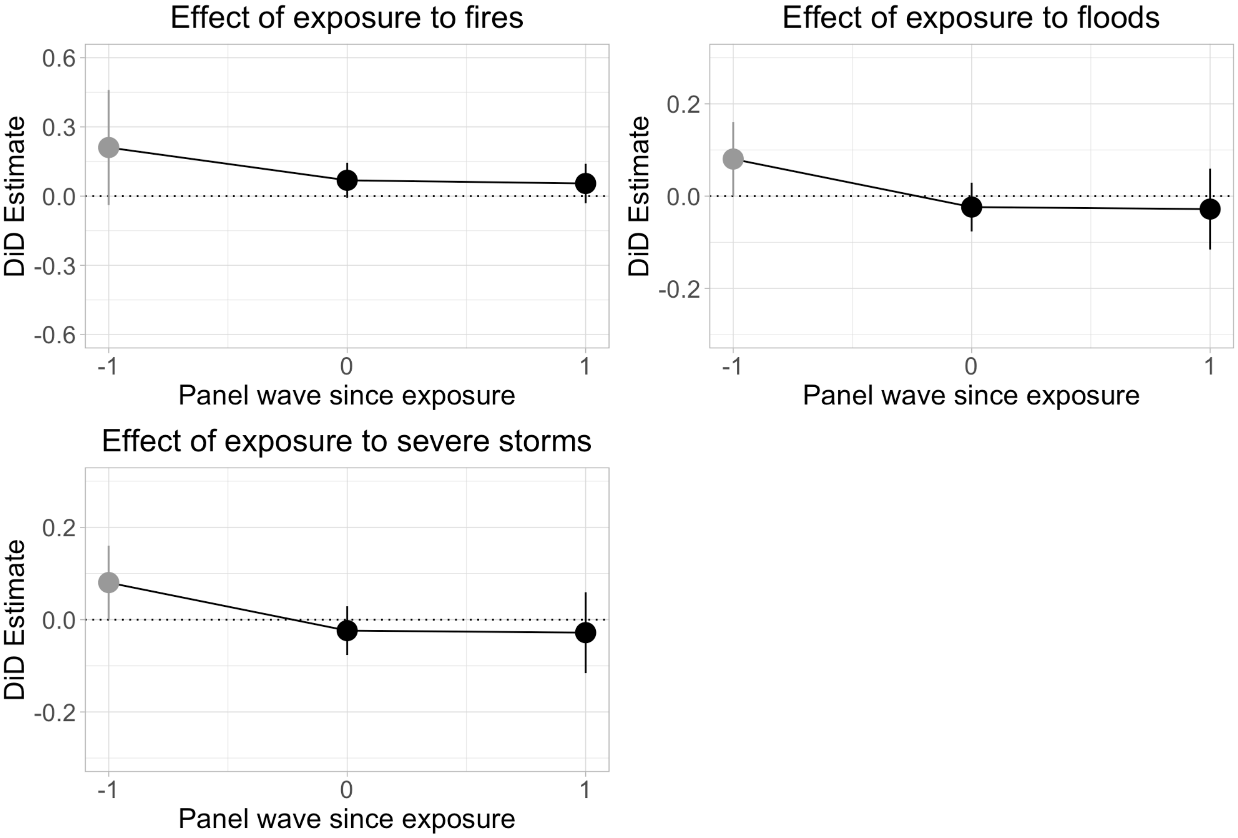

Figure A3: Effect of exposure to disasters on attitudes toward climate change. A length of exposure equal to 0 corresponds to the first or initial exposure to a disaster. A length of exposure equal to 1 corresponds to the first period after the initial exposure to a disaster. Conversely, a length of exposure equal to -1 refers to the first period before initial exposure. N = 28,128 (respondents-wave).

D Results in table format

Table A1: Effect of exposure to fires

Event time	Estimate	Std. Error	95% Conf. Int.
-1	-0.042	0.062	-0.182, 0.099
0	0.080	0.034	0.003,0.157
1	0.039	0.041	-0.054, 0.133

Table A2: Effect of exposure to floods

Event time	Estimate	Std. Error	95% Conf. Int.
-1	0.062	0.031	-0.010, 0.134
0	0.002	0.020	-0.043, 0.047
1	-0.017	0.029	-0.083, 0.049

Table A3: Effect of exposure to severe storms

Event time	Estimate	Std. Error	95% Conf. Int.
-1	0.032	0.052	-0.076, 0.139
0	-0.027	0.020	-0.068, 0.014
1	-0.028	0.023	-0.072, 0.019

Table A4: Effect of exposure to huricanes

Event time	Estimate	Std. Error	95% Conf. Int.
-1	0.027	0.086	-0.155,0.210
0	-0.026	0.024	-0.076,0.024
1	-0.022	0.030	-0.085,0.040

References

Buis, Alan. 2019. "Can Climate Affect Earthquakes, Or Are the Connections Shaky?" https://climate.nasa.gov/news/2926/can-climate-affect-earthquakes-or-are-the-connections-shaky/.

Cody, Emily M, Jennie C Stephens, James P Bagrow, Peter Sheridan Dodds and Christopher M Danforth. 2017. "Transitions in climate and energy discourse between Hurricanes Katrina and Sandy." *Journal of Environmental Studies and Sciences* 7(1):87–101.

Field, Christopher B, Vicente Barros, Thomas F Stocker and Qin Dahe. 2012. *Managing the risks of extreme events and disasters to advance climate change adaptation: special report of the intergovernmental panel on climate change*. Cambridge University Press.

Gariano, Stefano Luigi and Fausto Guzzetti. 2016. "Landslides in a changing climate." *Earth-Science Reviews* 162:227–252.

Hamilton, Lawrence C, Joel Hartter, Barry D Keim, Angela E Boag, Michael W Palace, Forrest R Stevens and Mark J Ducey. 2016. "Wildfire, climate, and perceptions in Northeast Oregon." *Regional environmental change* 16(6):1819–1832.

IPCC. 2018. *Summary for Policymakers. In: IPCC Special Report on the Ocean and Cryosphere in a Changing Climate*. IPCC Geneva.

Lang, Corey. 2014. "Do weather fluctuations cause people to seek information about climate change?" *Climatic change* 125(3-4):291–303.

Mann, Michael E and Kerry A Emanuel. 2006. "Atlantic hurricane trends linked to climate change." *Eos, Transactions American Geophysical Union* 87(24):233–241.

Marlon, Jennifer R, Patrick J Bartlein, Megan K Walsh, Sandy P Harrison, Kendrick J Brown, Mary E Edwards, Philip E Higuera, MJ Power, RS Anderson, Christy Briles et al. 2009. "Wildfire responses to abrupt climate change in North America." *Proceedings of the National Academy of Sciences* 106(8):2519–2524.

Masson-Delmotte, Valérie, Panmao Zhai, Hans-Otto Pörtner, Debra Roberts, Jim Skea, Priyadarshi R Shukla, Anna Pirani, W Moufouma-Okia, C Péan, R Pidcock et al. 2018. "Global warming of 1.5 C." *An IPCC Special Report on the impacts of global warming of* 1.

McBean, GA. 2005. Risk mitigation strategies for tornadoes in the context of climate change and development. In *Mitigation of natural hazards and disasters: International perspectives*. Springer pp. 25–34.

NASA. 2020. https://climate.nasa.gov/effects/.

NOAA. 2020. "Economics and Demographics." https://coast.noaa.gov/states/fast-facts/economics-and-demographics.html.

Spence, Alexa, Wouter Poortinga, Catherine Butler and Nicholas Frank Pidgeon. 2011. "Perceptions of climate change and willingness to save energy related to flood experience." *Nature climate change* 1(1):46.

Whitmarsh, Lorraine. 2008. "Are flood victims more concerned about climate change than other people? The role of direct experience in risk perception and behavioural response." *Journal of risk research* 11(3):351–374.
