# Supplementary material for: The effect of different extreme weather events on attitudes toward climate change
Source: PLoS One. 2024 May 15;19(5):e0300967. doi: 10.1371/journal.pone.0300967 (PMC11095695; doi:10.1371/journal.pone.0300967)
Supplement: Attachment — Submitted filename: memo_revisions.pdf [file pone.0300967.s003.pdf]

Response Memo:
The Effect of Different Extreme Weather Events on Attitudes Toward Climate Change
PONE-D-23-27737

Reply to Editor

Thank you very much for the opportunity to revise and resubmit our article to PLOS ONE. We appreciate the reviewers' useful and thoughtful suggestions. We have attempted to address all the comments, which we believe have greatly improved our manuscript.

In this memo, we detail the specific responses to each reviewer point by point and explain how we have addressed their suggestions in the revised manuscript or appendix. Please let us know if there are any further concerns or questions.

Reply to Reviewer 1

1. *Literature Review: The paper requires a more comprehensive review of the existing literature concerning the influence of extreme weather events (EWEs) on behavior and attitudes. While you reference studies such as Sloggy et al. (2023), Whitmarch (2008), and Spence et al. (2011), more information is needed to understand their methodologies, findings, and the specific EWEs they investigated. Additional aspects are here also what behavioral dimension they examined (behavior, attitudes, support for specific or general measures, etc.) and the timing they assess the impact of the EWE. Additionally, several studies that discuss behavioral insights in this domain, such as e.g. Gärtner and Schön (2021) are relevant to your research question but are not mentioned at all.*

Thank you for the comments and suggestions. We have included an additional section in the paper to better review relevant literature, and have incorporated your suggestions to describe their methodologies, findings, and the specific EWEs the included studies examine.

2. *Research Design: Essential details about your research design are missing. such as: - the nature of the FEMA dataset, who gets surveyed, when, how, and with which survey technique?*

   The FEMA data provides a summary of major disasters and other emergencies beginning in 1953, including severe flooding, volcanic eruptions, landslides, and chemical waste exposures. Data is provided at the county-level and includes the start date of each disaster declaration as well as an incident type (e.g., flood, tornado).

   Regarding the survey data, the CCES panel study is an online survey conducted by YouGov. The sampling method is matched random sample, which attempts to mimic a representative sample from non-randomly selected pools of respondents. This type of design is ideally suited for online panels (Ansolabehere and Schaffner 2015).

   We have included both explanations in the new version of the manuscript.

3. *A clarification of the meaning of "eventually treated"?*

   Our design is based on comparing a control and exposed group over time. The control group is composed of never-treated respondents (i.e., subjects that were not exposed to a disaster) and the exposed group of subjects that were exposed to a disaster. Importantly, exposure to a disaster can happen at different times, such as during the first, second, or third wave of the panel study. Subjects who live in an exposed county but before exposure are called eventually exposed or eventually treated. For example, a respondent living in a county exposed to a disaster during the 2012 wave would be considered eventually exposed during the 2010 wave.

4. *Clarification/Justification why data only from 2010, 2012, and 2014 is analyzed. More*

recent data would have been certainly be better (see point below on validity)

We acknowledge that more data, including more recent data, would have been ideal to study trends over time. However, the CCES Panel Study with relevant measures for the purposes of this study only included three waves: 2010, 2012, and 2014. Therefore, our design is restricted by the structure of the panel study.

5. *Elaboration of the statement "estimates are aggregated at the length of exposure". You mean the length of the exposure of a flood that e.g. took place over 3 days?*

   An event study or dynamic DiD uses a staggered treatment, meaning that when a unit is considered exposed, it stays in that group and does not go back to the control group. Therefore, this allows us to estimate the effect of immediate or initial exposure to a disaster and to estimate the effect of one year after initial exposure to a disaster. Effects are aggregated, meaning that someone might be exposed to a disaster for the first time in 2012 or 2014, but we provide the effect of initial exposure, not the effect of exposure in 2012 or 2014.

6. *How do you account for then for factors like the frequency of exposure and time since exposure - that very likely shape the results?*

   This is a great point. Our design considered someone as exposed if a disaster happened in their county the year before the survey implementation, which means that there is a gap between exposure and the survey. Based on previous research showing how disasters can have long-lasting effects(Bechtel and Hainmueller 2011), we expect that a small gap should not have a meaningful effect on the main findings. Regarding the frequency, the Callaway and Sant'anna DID estimator we use in the manuscript can only be implemented using a binary indicator of exposure, limiting our capacity to capture the effect based on exposure doses.

7. *Time Analysis: Since you use a binary indicator of exposure to EWE, a complementary analysis that takes into account the time since exposure is needed. This would allow us*

to see whether, depending on the specific EWE type, there was an effect at all and how it deteriorated.

That is exactly what the dynamic difference-in-differences is doing. We acknowledge this might not have been well explained in the paper. For each disaster type, we provided three different point estimates. The first reported in grey represents the pre-treatment results, which are computed using the never-treated and the eventually-treated subjects (i.e., evidence for the parallel trend assumption). The second reported in black represents the effect of initial or immediate exposure. The third, also reported in black, represents the effect one year after initial exposure, meaning that those results take into account the time since exposure.

8. *Discussion on the Relevance of Findings: Include a discussion about the relevance and up-to-dateness of your findings, particularly in the context of political discussions and climate action, and given your data is 10 years + old.*

   We acknowledge that more data, including more recent data, would have been ideal to study trends over time. However, we believe there are several advantages to examining the impact of extreme weather events on public opinion during the time period we study. For one, it offers insights prior to the COVID-19 pandemic, which could have shifted priorities and the relative impact of weather events. This data is also prior to the Trump Administration, which significantly rolled back many existing measures to address climate change and may have created a kind of backlash effect. Finally, it's important to recall that extreme weather events are anticipated to become more frequent and severe as climate change progresses. Understanding how extreme weather events may have influenced climate attitudes prior to such environmental and political changes could be particularly useful when compared with more recent data, thereby offering insights into how extreme weather events influence attitudes amidst other pressing developments and when extreme weather is a more common occurrence.

9. *Minor Suggestions: - Update the references in your introduction related to climate variability with more recent sources. Much happened here in the last 10 years! - Ensure that Figure 1 accurately represents the timing of EWEs and provide clarity on this aspect within the figure description. As far as I understood t=0 is not the EWE but the point of the following survey measurement, which can be many months after. - Specify the abbreviation "FEMA" when it is first used. - Include information on the nine disaster types in the main text.*

   We have addressed these comments and improved Figure 1. Thanks for the suggestions.

Reply to Reviewer 2

1. *Introduction: The Introduction offers only a cursory treatment of the relevant literature and relies on outdated citations. It would be useful to add a Background Literature section to expand on these important conceptual frameworks and related findings. For example: P. 1 In the first couple of sentences, more recent citations would be useful – most are over a decade old. The Smith (2020) citation is a good one, although there is a more recent version (Smith, 2023). P. 1 the discussion of the connection between "mass attitudes" and policy change is all of one sentence, even though there has been considerable treatment of these concepts, including the focusing events literature (e.g., Birkland, 2006). Relatedly, the discussion of the theorized relationship between extreme events, public opinion and policy change includes only one reference (Howe et al., 2019) - which is not even the most current review – despite important seminal articles (e.g., Weber, 2010, 2013), more recent reviews (e.g., Brügger, 2020; Giordono et al., 2020; Howe, 2021) and other empirical contributions to a now-sizable literature (e.g., Boudet et al., 2019; Giordono et al., 2023; Hazlett & Mildenberger, 2020; Zanocco et al., 2019) .*

   Thank you for the comments and suggestions. We have included an additional section in the paper to better review relevant literature.

2. *The research design - specifically the analytic approach – is appropriate and it's great to see a dynamic difference-in-difference/event study approach applied to panel data to study these questions. However, the narrative needs considerable clarification, and in some places, additional citation. This is especially true for the non-technical reader, who is likely familiar with traditional diff-in-diff, but not necessarily with this approach. Finally, as stated earlier, the data are over a decade old, so the implications of recent changes should be discussed*

   Thanks for the comment. We have substantially edited the research design to better explain what is a dynamic difference-in-differences and how it works. Also, we have also improved the explanation of the main findings. Finally, we engage with concerns about the time of the panel survey data in our answer to question 8 from Reviewer 1.

3. *P. 2 - In the second sentence, there are other relevant citations that could be useful here (more than just a self-cite). See, for example, some of the suggestions above. Seems especially important to describe the strong evidence about the consistent influence of political ideology/party. Also note that these first three sentences, and related citations, would also probably be more appropriate in a thorough Background Literature section. I also wonder whether it would be useful to add hypotheses.*

   Thank you for the comments and suggestions. We have included an additional section in the paper to better review relevant literature. Also, we see this paper as an empirical exercise to learn about the causal effects of different types of disasters. Therefore, we do not express clear expectations through hypotheses.

4. *P. 3 - It might be useful to present both the estimating equation and the parallel trends assumption equation here. On the other hand, it could be alienating to the non-technical reader. Seems like an editorial call.*

   We have improved the explanation of how each result is computed as a way to address this comment. Thanks for the suggestion.

5. *P. 3 - Please discuss the inclusion of covariates (e.g., why you included them, even though DiD should, in theory, "difference out" static characteristics). Also indicate which covariates you included.*

   Covariates were included for efficiency reasons, so they help to reduce standard errors. Findings are consistent when covariates are not included. In the new version, we report the main findings without covariates in the manuscript and with covariates in Appendix B. For covariates, we used education, gender, and age in 2010, which are placebo covariates since those should not be affected by exposure to a disaster.

6. *P. 3 - Do you use cluster-robust standard errors? Or does the "doubly-robust" estimator (Sant'Anna & Zhao, 2020) negate the need for cluster-robust standard errors?*

   We use bootstrapped-based standard errors, which is recommended when exposure happens in a cluster design (Cameron 2008). We clarify this in the manuscript.

7. *P. 4 - it is standard to present your data source first, followed by your analytic approach. And in this case, it is especially important, given that you're working with panel data. I would strongly recommend switching those sections and editing as necessary.*

   We have followed this advice. Thank you.

8. *P. 4 - I found the Data section (2.2.) a little hard to follow and would like to see more detailed information, as follows: I would start with a quick reference to both data sources and/or include a table that shows all variables used in the analysis (including the covariates), along with the source, variable operationalization and descriptive stats.*

   We have added citations for both data sources.

9. *Regarding the disaster declaration variables - are these state-level declarations? So someone in a specific state that received federal disaster declaration is assumed to have been "exposed", correct? Or did you use some other type of geospatial matching process to identify exposure? Please clarify, and offer any implications for findings (seems like there's likely*

to be considerable measurement error, especially for some event types - what are the likely implications for findings?).

These are county-level declarations. FEMA is official data and has been used in previous research to measure exposure to EWEs. This data has been used by other scholars like Sloggy et al. (2021) to study the influence of disasters on climate opinions. However, we acknowledge that this might involve some measurement error. Since the dynamic DID is based on a binary measure, using the official declaration of a disaster works as an empirical strategy. We encourage further research to use geospatial or climatological data as a continuous variable of exposure to compare that with our findings.

10. *Relatedly, do we know when in the prior year that the respondent was exposed? Presumably we know when the survey was conducted and when the event occurred. With a binary measure of exposure, you're lumping everyone in the same group, whether their exposure was 1 month prior or 12 months prior, correct?*

    Our design considered someone as exposed if a disaster happened in their county the year before the survey implementation, which means that there is a gap between exposure and the survey. Based on previous research showing how disasters can have long-lasting effects (Bechtel and Hainmueller 2011,), we expect that a small gap should not have a meaningful effect on the main findings. Regarding the frequency, the Callaway and Sant'anna DID estimator we use in the manuscript can only be implemented using a binary indicator of exposure, limiting our capacity to capture the effect based on exposure doses.

11. *I had trouble understanding the alignment between FEMA declaration data and event exposure. You indicate that you construct your indicator of exposure based on one year before the survey year. So for the panel wave implemented in 2010, you use FEMA data for 2009. And presumably for the 2012 panel, you use FEMA data for 2011. Does that mean you disregard potential exposure in 2010?*

    That is correct. For a survey implemented in 2010, we use exposure in 2009. Our expectation

is the effect of exposure to a disaster on concerns about climate change will take some time to crystallize since disaster victims usually emphasize concerns about their living conditions just after a disaster rather than paying attention to public or collective concerns (Visconti, 2021), such as the impact of climate change. However, as a robustness check, we use events that happened the same year as the survey but before its implementation (see Appendix C). That approach does not change our main conclusions.

12. *A little more precision around language might help make your process more transparent. For example, "respondents who are exposed in the first wave (i.e., always treated" presumably means respondents who were exposed prior to the first survey wave, rather than "in" it.*

    Correct, always treated are subjects that, during the first wave, are considered exposed, meaning that were exposed in the previous year. We clarify that in the manuscript.

13. *More generally, a visual of the alignment between the FEMA and panel data might be helpful here. In particular, one that shows the relationship between the each declaration year, panel wave and subject group. I could see a flow chart or table performing this function.*

    We have better explained the structure of the panel and the FEMA data in the manuscript. We hope that those changes have helped to clarify the Reviewer's comments.

14. *P. 5 - As noted above, I'm concerned about the age of the data. That said, I have some concerns about basing conclusions on data that is approximately a decade old. The authors need to acknowledge this limitation, and describe how older data are relevant to our understanding of current "mass attitudes," and potential for policy change, given that the American public is currently operating at a higher baseline with respect to beliefs - almost 75% of the American public believes in global warming (Leiserowitz et al., 2023) and experiencing extreme events with a higher frequency and severity (Smith, 2020) than they did a decade ago?*

    We acknowledge that more data, including more recent data, would have been ideal to study

trends over time. However, we believe there are several advantages to examining the impact of extreme weather events on public opinion during this time period. For one, it offers insights prior to the COVID-19 pandemic, which could have shifted priorities and the relative impact of weather events. This data is also prior to the Trump Administration, which significantly rolled back many existing measures to address climate change and may have created a kind of backlash effect. Finally, it's important to recall that extreme weather events are anticipated to become more frequent and severe as climate change progresses. Understanding how extreme weather events may have influenced climate attitudes prior to such environmental and political changes could be particularly useful when compared with more recent data, thereby offering insights into how extreme weather events influence attitudes amidst other pressing developments and when extreme weather is a more common occurrence.

15. *P. 5 - It's unfortunate that the CCES questions are double-barreled (asking about beliefs and action in the same question stem and response options). Please discuss the implications for your results.*

    It is unfortunate the measures are double-barreled. This is an issue other scholars like Konisky et al. 2016 have faced, who draw on the same CCES data. We have acknowledged this limitation in the manuscript.

16. *P. 5 - On p. 3, you referred to the education, gender and age as the covariates that were incorporated into the analysis. What about political affiliation/orientation? That is a key predictor of climate change beliefs, so it seems important to account for it. If the data simply aren't available, then that gap should be acknowledged and potential implications explained, or else cite evidence that political affiliation doesn't typically change over time and therefore will be differenced out in this study.*

    We decided only to include placebo covariates, or in other words, subjects' characteristics that should not be affected by exposure to a disaster. We made this decision since we do not have pretreatment characteristics (covariates are captured at the same time as exposure).

However, in Appendix B, we included the results when controlling for a binary indicator of identifying as Republican or Democrat, and the conclusions of our study do not change. We use partisanship in a different analysis because this might not necessarily be a placebo covariate since exposure to disaster can affect different political outcomes.

17. *While the results appear to be accurate and relevant, the Results section could also use some additional attention: P. 5 – It is standard to provide descriptive statistics for all variables included in the main analysis. See for example, Table 2 in Calloway and Sant'Anna (2020), which is cited in your study. They could either be placed in a broader table in the Data section, as suggested above, or in the Results section. This seems particularly important for understanding the magnitude of the effects.*

    Thanks. We have included a descriptive statistics table in the new version of the manuscript.

18. *P. 5 - In the third sentence, you describe the three groups that correspond to the post-exposure analysis, or time after initial event exposure (-1, 0 and 1). However, I'm still having a hard time understanding what happens to the experience during survey "gap years" (i.e., the years between survey waves - for example, 2011).*

    We have improved that figure. The x-axis reflects the year to/from exposure to a disaster. Thanks for the suggestion.

19. *P. 6 - Figure 1a shows the effect estimates, and I like seeing the visual. However, it is standard to also present the regression results in a table, either in the main text or an appendix.*

    We now include the regression table in Appendix D. Thanks.

20. *P. 6 - It would also be useful to graph the actual data for the various subject groups to give the reader an opportunity to "eyeball" the pre-treatment trend. If you are space-constrained, such a graph could be shown in an appendix. Along those lines, please reiterate to the reader that Figure 1a graphs the effects, not the actual data.*

Correct, Figure 1 provides the effects, not the data. We now provide a descriptive statistics table in the manuscript. We hope that helps to clarify the reviewer's concerns.

21. *P. 6 - In figure 1a, the dotted line represents the event time, and the survey was done within 12 months of the event, correct? So the "treatment" occurs between -1 and 0, but we don't know exactly when... this graph makes it look like it occurred just prior to time 0. Doesn't seem like an accurate visualization. Perhaps you should shade that space on the graph to represent the possible range of the event exposure period, or simply take out the dotted line altogether.*

    We agree. We have removed the dotted line to improve the visualization. Thanks.

22. *P. 6 - It might be more precise (and clear) to label the X-axis as "Time to/since event," rather than "Event time".*

    We corrected that. Thanks.

23. *P. 6 - You indicated earlier that covariates were included. Were any of them significant? (I wouldn't expect them to be significant, given the research design, but it seems important to add this information). Alternatively, this information could be part of a regression table.*

    Covariates are included for efficiency reasons. As a result, we prefer not to interpret them following Keele et al. (2020) recommendation.

24. *P. 6 - Please interpret the findings for the non-technical reader. For example, is the magnitude of the statistically significant effects of fire exposure meaningful? Maybe calculate effect size?*

    Thanks. Now, we also provide that finding in standard deviation units to facilitate its interpretation. In the manuscript, we say: "When using a standardized version of the outcome, first exposure to fire (event time = 0) increases the outcome by 0.06 standard deviation units, which is a small-sized effect."

25. *P. 6 - What does the N=28,128 mean? I am presuming that you conducted each event-specific analysis separately, using the same control group but different treatment groups. If that is the case, then shouldn't the N be different for each treatment group?*

    28,128 are respondents-wave; we clarify this in the manuscript. They represent the total number of observations available. The reviewer is correct that each analysis in Figure 1 is based on subsets of this large dataset, such as comparing never exposed and eventually exposed (first point estimate in the figure).

26. *Conclusion: The Conclusion seems thin, with respect to study limitations, implications for political processes and/or policy, and relevant other references. Specifically: Please add a paragraph describing the limitations of the study. The implications of the main findings for fire exposure should potentially be unpacked a little more, with reference to recent work relating to fire exposure, public opinion and public policy. In addition to the works cited earlier, there are several recent articles that examine support for, and effects of, public safety power shutoffs on beliefs and attitudes that could also be cited (e.g., Guliasi, 2021; Mildenberger et al., 2022; Zanocco et al., 2021, 2022).*

    Thank you for these helpful suggestions. We have incorporated a brief discussion about the implications for fire exposure. While we are excited about the contributions of this paper, we have also included a statement about its limitations and potential areas to explore based on our findings.
