# Supplementary material for: The effect of different extreme weather events on attitudes toward climate change
Source: PLoS One. 2024 May 15;19(5):e0300967. doi: 10.1371/journal.pone.0300967 (PMC11095695; doi:10.1371/journal.pone.0300967)
Supplement: Attachment — Submitted filename: PONE-D-23-27737_RefereeReport.docx [file pone.0300967.s002.docx]

**Manuscript ID:** PONE-D-23-27737

**Manuscript Title:** The Effect of Different Extreme Weather Events on Attitudes Toward Climate Change

**Manuscript Summary:** This study uses panel survey data in the context of a Difference-in-Difference event study analysis to estimate the effects of exposure to a selection of extreme events on climate change beliefs.

In the sections below, I offer several high-level thoughts, followed by specific bulleted questions or comments.

**Overall comments:**

This paper is well-motivated and the methods are relevant. However, there are considerable opportunities for providing more detailed information about background and methods, as well as connecting the study to the current context.

**Motivation/significance:**

The paper is well-motivated and fits into the nascent, but growing, literature on the connection between extreme weather events and climate change beliefs, attitudes and policy support. The methods are also appropriate, and to my knowledge, are the first application of diff-in-diff event study (and related inferences) to these questions. In particular, the examination of different event types is an important contribution. Finally, the data are somewhat dated, which affects the degree to which we can apply the findings to current context.

**Introduction:**

The Introduction offers only a cursory treatment of the relevant literature and relies on outdated citations. It would be useful to add a Background Literature section to expand on these important conceptual frameworks and related findings. For example:

- P. 1 – In the first couple of sentences, more recent citations would be useful – most are over a decade old. The Smith (2020) citation is a good one, although there is a more recent version (Smith, 2023).
- P. 1 – the discussion of the connection between “mass attitudes” and policy change is all of one sentence, even though there has been considerable treatment of these concepts, including the focusing events literature (e.g., Birkland, 2006). Relatedly, the discussion of the theorized relationship between extreme events, public opinion and policy change includes only one reference (Howe et al., 2019) – which is not even the most current review -- despite important seminal articles (e.g., Weber, 2010, 2013), more recent reviews (e.g., Brügger, 2020; Giordono et al., 2020; Howe, 2021) and other empirical contributions to a now-sizable literature (e.g., Boudet et al., 2019; Giordono et al., 2023; Hazlett & Mildenberger, 2020; Zanocco et al., 2019) .

**Research Design:**

The research design – specifically the analytic approach -- is appropriate and it’s great to see a dynamic difference-in-difference/event study approach applied to panel data to study these questions. However, the narrative needs considerable clarification, and in some places, additional citation. This is especially true for the non-technical reader, who is likely familiar with traditional diff-in-diff, but not necessarily with this approach. Finally, as stated earlier, the data are over a decade old, so the implications of recent changes should be discussed. Specific comments include:

- P. 2 – In the second sentence, there are other relevant citations that could be useful here (more than just a self-cite). See, for example, some of the suggestions above. Seems especially important to describe the strong evidence about the consistent influence of political ideology/party. Also note that these first three sentences, and related citations, would also probably be more appropriate in a thorough Background Literature section. I also wonder whether it would be useful to add hypotheses.
- P. 3 – It might be useful to present both the estimating equation and the parallel trends assumption equation here. On the other hand, it could be alienating to the non-technical reader. Seems like an editorial call.
- P. 3 – Please discuss the inclusion of covariates (e.g., why you included them, even though DiD should, in theory, “difference out” static characteristics). Also indicate which covariates you included.
- P. 3 – Do you use cluster-robust standard errors? Or does the “doubly-robust” estimator (Sant’Anna & Zhao, 2020) negate the need for cluster-robust standard errors?
- P. 4 – it is standard to present your data source first, followed by your analytic approach. And in this case, it is especially important, given that you’re working with panel data. I would strongly recommend switching those sections and editing as necessary.
- P. 4 – I found the Data section (2.2.) a little hard to follow and would like to see more detailed information, as follows:
  - I would start with a quick reference to both data sources and/or include a table that shows all variables used in the analysis (including the covariates), along with the source, variable operationalization and descriptive stats.
  - Regarding the disaster declaration variables – are these state-level declarations? So someone in a specific state that received federal disaster declaration is assumed to have been "exposed", correct? Or did you use some other type of geospatial matching process to identify exposure? Please clarify, and offer any implications for findings (seems like there’s likely to be considerable measurement error, especially for some event types – what are the likely implications for findings?).
  - Relatedly, do we know when in the prior year that the respondent was exposed? Presumably we know when the survey was conducted and when the event occurred. With a binary measure of exposure, you’re lumping everyone in the same group, whether their exposure was 1 month prior or 12 months prior, correct?
  - I had trouble understanding the alignment between FEMA declaration data and event exposure. You indicate that you construct your indicator of exposure based on one year before the survey year. So for the panel wave implemented in 2010, you use FEMA data for 2009. And presumably for the 2012 panel, you use FEMA data for 2011. Does that mean you disregard potential exposure in 2010?
  - A little more precision around language might help make your process more transparent. For example, “respondents who are exposed in the first wave (i.e., always treated” presumably means respondents who were exposed **prior** to the first survey wave, rather than “in” it.
  - More generally, a visual of the alignment between the FEMA and panel data might be helpful here. In particular, one that shows the relationship between the each declaration year, panel wave and subject group. I could see a flow chart or table performing this function.
- P. 5 – As noted above, I’m concerned about the age of the data. That said, I have some concerns about basing conclusions on data that is approximately a decade old. The authors need to acknowledge this limitation, and describe how older data are relevant to our understanding of current “mass attitudes,” and potential for policy change, given that the American public is currently operating at a higher baseline with respect to beliefs – almost 75% of the American public believes in global warming (Leiserowitz et al., 2023) and experiencing extreme events with a higher frequency and severity (Smith, 2020) than they did a decade ago?
- P. 5 – It’s unfortunate that the CCES questions are double-barreled (asking about beliefs and action in the same question stem and response options). Please discuss the implications for your results.
- P. 5 – On p. 3, you referred to the education, gender and age as the covariates that were incorporated into the analysis. What about political affiliation/orientation? That is a key predictor of climate change beliefs, so it seems important to account for it. If the data simply aren’t available, then that gap should be acknowledged and potential implications explained, or else cite evidence that political affiliation doesn’t typically change over time and therefore will be differenced out in this study.

**Results:**

While the results appear to be accurate and relevant, the Results section could also use some additional attention:

- P. 5 -- It is standard to provide descriptive statistics for all variables included in the main analysis. See for example, Table 2 in Calloway and Sant’Anna (2020), which is cited in your study. They could either be placed in a broader table in the Data section, as suggested above, or in the Results section. This seems particularly important for understanding the magnitude of the effects.
- P. 5 – In the third sentence, you describe the three groups that correspond to the post-exposure analysis, or time after initial event exposure (-1, 0 and 1). However, I’m still having a hard time understanding what happens to the experience during survey “gap years” (i.e., the years between survey waves – for example, 2011).
- P. 6 – Figure 1a shows the effect estimates, and I like seeing the visual. However, it is standard to also present the regression results in a table, either in the main text or an appendix.
- P. 6 -- It would also be useful to graph the actual data for the various subject groups to give the reader an opportunity to “eyeball” the pre-treatment trend. If you are space-constrained, such a graph could be shown in an appendix. Along those lines, please reiterate to the reader that Figure 1a graphs the effects, not the actual data.
- P. 6 – In figure 1a, the dotted line represents the event time, and the survey was done within 12 months of the event, correct? So the "treatment" occurs between -1 and 0, but we don't know exactly when... this graph makes it look like it occurred just prior to time 0. Doesn't seem like an accurate visualization. Perhaps you should shade that space on the graph to represent the possible range of the event exposure period, or simply take out the dotted line altogether.
- P. 6 -- It might be more precise (and clear) to label the X-axis as “Time to/since event,” rather than “Event time”.
- P. 6 – You indicated earlier that covariates were included. Were any of them significant? (I wouldn’t expect them to be significant, given the research design, but it seems important to add this information). Alternatively, this information could be part of a regression table.
- P. 6 – Please interpret the findings for the non-technical reader. For example, is the magnitude of the statistically significant effects of fire exposure meaningful? Maybe calculate effect size?
- P. 6 – What does the N=28,128 mean? I am presuming that you conducted each event-specific analysis separately, using the same control group but different treatment groups. If that is the case, then shouldn’t the N be different for each treatment group?

**Conclusion:**

The Conclusion seems thin, with respect to study limitations, implications for political processes and/or policy, and relevant other references. Specifically:

- Please add a paragraph describing the limitations of the study.
- The implications of the main findings for fire exposure should potentially be unpacked a little more, with reference to recent work relating to fire exposure, public opinion and public policy. In addition to the works cited earlier, there are several recent articles that examine support for, and effects of, public safety power shutoffs on beliefs and attitudes that could also be cited (e.g., Guliasi, 2021; Mildenberger et al., 2022; Zanocco et al., 2021, 2022).

**References**

Birkland, T. A. (2006). *Lessons of disaster: Policy change after catastrophic events*. Georgetown University Press. http://ebookcentral.proquest.com/lib/osu/detail.action?docID=547783

Boudet, H., Giordono, L., Zanocco, C., Satein, H., & Whitley, H. (2019). Event attribution and partisanship shape local discussion of climate change after extreme weather. *Nature Climate Change*, 1–8. https://doi.org/10.1038/s41558-019-0641-3

Brügger, A. (2020). Understanding the psychological distance of climate change: The limitations of construal level theory and suggestions for alternative theoretical perspectives. *Global Environmental Change*, *60*, 102023. https://doi.org/10.1016/j.gloenvcha.2019.102023

Giordono, L., Boudet, H., & Gard-Murray, A. (2020). Local adaptation policy responses to extreme weather events. *Policy Sciences*. https://doi.org/10.1007/s11077-020-09401-3

Giordono, L., Siddiqi, M. U. A., Stelmach, G., Zanocco, C., Flora, J., & Boudet, H. (2023). *Trial by Fire: Support for Mitigation and Adaptation Policy after the 2020 Oregon Wildfires*.

Guliasi, L. (2021). Toward a political economy of public safety power shutoff: Politics, ideology, and the limits of regulatory choice in California. *Energy Research & Social Science*, *71*, 101842. https://doi.org/10.1016/j.erss.2020.101842

Hazlett, C., & Mildenberger, M. (2020). Wildfire Exposure Increases Pro-Environment Voting within Democratic but Not Republican Areas. *American Political Science Review*, 1–7. https://doi.org/10.1017/S0003055420000441

Howe, P. D. (2021). Extreme weather experience and climate change opinion. *Current Opinion in Behavioral Sciences*, *42*, 127–131. https://doi.org/10.1016/j.cobeha.2021.05.005

Howe, P. D., Marlon, J. R., Mildenberger, M., & Shield, B. S. (2019). How will climate change shape climate opinion? *Environmental Research Letters*, *14*(11), 113001. https://doi.org/10.1088/1748-9326/ab466a

Leiserowitz, A., Maibach, E. W., Rosenthal, S. A., Kotcher, J. E., Lee, S., Verner, M., Ballew, M. T., Carman, J. P., Myers, T. A., Goldberg, M. H., Badullovich, N., & Marlon, J. R. (2023). *Climate Change in the American Mind: Beliefs and Attitudes (Spring 2023)*. Yale University and George Mason University. https://climatecommunication.yale.edu/wp-content/uploads/2023/09/climate-change-american-mind-beliefs-attitudes-spring-2023.pdf

Mildenberger, M., Howe, P. D., Trachtman, S., Stokes, L. C., & Lubell, M. (2022). The effect of public safety power shut-offs on climate change attitudes and behavioural intentions. *Nature Energy*, 1–8. https://doi.org/10.1038/s41560-022-01071-0

Sant’Anna, P. H. C., & Zhao, J. (2020). Doubly robust difference-in-differences estimators. *Journal of Econometrics*, *219*(1), 101–122. https://doi.org/10.1016/j.jeconom.2020.06.003

Smith, A. B. (2020). *U.S. Billion-dollar Weather and Climate Disasters, 1980—Present (NCEI Accession 0209268)* [dataset]. NOAA National Centers for Environmental Information. https://doi.org/10.25921/STKW-7W73

Smith, A. B. (2023). *2022 U.S. billion-dollar weather and climate disasters in historical context | NOAA Climate.gov*. National Oceanic and Atmospheric Administration. http://www.climate.gov/news-features/blogs/2022-us-billion-dollar-weather-and-climate-disasters-historical-context

Weber, E. U. (2010). What shapes perceptions of climate change? *WIREs Climate Change*, *1*(3), 332–342. https://doi.org/10.1002/wcc.41

Weber, E. U. (2013). Seeing is believing. *Nature Climate Change*, *3*(4), 312–313. https://doi.org/10.1038/nclimate1859

Zanocco, C., Boudet, H., Nilson, R., & Flora, J. (2019). Personal harm and support for climate change mitigation policies: Evidence from 10 U.S. communities impacted by extreme weather. *Global Environmental Change*, *59*, 101984. https://doi.org/10.1016/j.gloenvcha.2019.101984

Zanocco, C., Flora, J., Rajagopal, R., & Boudet, H. (2021). When the lights go out: Californians’ experience with wildfire-related public safety power shutoffs increases intention to adopt solar and storage. *Energy Research & Social Science*, *79*, 102183. https://doi.org/10.1016/j.erss.2021.102183

Zanocco, C., Stelmach, G., Giordono, L., Flora, J., & Boudet, H. (2022). Poor Air Quality during Wildfires Related to Support for Public Safety Power Shutoffs. *Society & Natural Resources*, *0*(0), 1–15. https://doi.org/10.1080/08941920.2022.2041138
